# Effective Synchronization of EEG and EMG for Mobile Brain/Body Imaging in Clinical Settings

**DOI:** 10.3389/fnhum.2017.00652

**Published:** 2018-01-11

**Authors:** Fiorenzo Artoni, Annalisa Barsotti, Eleonora Guanziroli, Silvestro Micera, Alberto Landi, Franco Molteni

**Affiliations:** ^1^The BioRobotics Institute, Scuola Superiore Sant’Anna, Pisa, Italy; ^2^Translational Neural Engineering Laboratory, Center for Neuroprosthetics and Institute of Bioengineering, EPFL, Lausanne, Switzerland; ^3^Department of Information Engineering, University of Pisa, Pisa, Italy; ^4^Valduce Hospital, Villa Beretta Rehabilitation Center, Costa Masnaga, Italy

**Keywords:** Mobile Brain/body Imaging, MoBI, EEG, EMG, synchronization, jitter, lab streaming layer

## Abstract

Mobile Brain/Body Imaging (MoBI) is rapidly gaining traction as a new imaging modality to study how cognitive processes support locomotion. Electroencephalogram (EEG) and electromyogram (EMG), due to their time resolution, non-invasiveness and portability are the techniques of choice for MoBI, but synchronization requirements among others restrict its use to high-end research facilities. Here we test the effectiveness of a technique that enables us to achieve MoBI-grade synchronization of EEG and EMG, even when other strategies (such as Lab Streaming Layer (LSL)) cannot be used e.g., due to the unavailability of proprietary Application Programming Interfaces (APIs), which is often the case in clinical settings. The proposed strategy is that of aligning several spikes at the beginning and end of the session. We delivered a train of spikes to the EEG amplifier and EMG electrodes every 2 s over a 10-min time period. We selected a variable number of spikes (from 1 to 10) both at the beginning and end of the time series and linearly resampled the data so as to align them. We then compared the misalignment of the “middle” spikes over the whole recording to test for jitter and synchronization drifts, highlighting possible nonlinearities (due to hardware filters) and estimated the maximum length of the recording to achieve a [−5 to 5] ms misalignment range. We demonstrate that MoBI-grade synchronization can be achieved within 10-min recordings with a 1.7 ms jitter and [−5 5] ms misalignment range. We show that repeated spike delivery can be used to test online synchronization options and to troubleshoot synchronization issues over EEG and EMG. We also show that synchronization cannot rely only on the equipment sampling rate advertised by manufacturers. The synchronization strategy described can be used virtually in every clinical environment, and may increase the interest among a broader spectrum of clinicians and researchers in the MoBI framework, ultimately leading to a better understanding of the brain processes underlying locomotion control and the development of more effective rehabilitation approaches.

## Introduction

The ability to walk independently is fundamental for the execution of daily life activities. Brain injuries (e.g., stroke) can cause motor damage comprising locomotion impairment with a negative impact on the quality of life. Thus, great effort is put into restoring walking in people with brain damage; in order to get a deeper understanding of cortical involvement during walking it is necessary to develop models that represent cortical activities in relation to human walking patterns. It is known that the cortex proactively controls voluntary and precise movements and is involved only in “high-level” motor planning (e.g., gait initiation, addressing obstacles, etc.). Its involvement during ambulation tasks however is only hypothesized because of the limits of available techniques (Marple-Horvat and Criado, 1999; Beloozerova and Sirota, 2003; Marigold et al., 2011; Drew and Marigold, 2015).

Until very recently, several technical constraints restricted ambulation studies to motor imagery (Schlögl et al., 2005), resting periods just before/just after exercise (Gutmann et al., 2015), detection of movement intentions (Bai et al., 2007) or other static tasks (e.g., reaching/grasping (Hammon et al., 2008)), leaving out of the analysis any movement-related sensory information and path integration aspect. The electroencephalogram (EEG), contrary to NIRS or fMRI, is portable, non-invasive, easy to mount, has fast time scale and it is becoming the technique of choice for brain imaging in humans in the rapidly emerging framework of Mobile Brain/Body Imaging (MoBI; Gramann et al., 2011, 2014). Similarly, surface electromyogram (EMG) allows muscle activity recording and analysis with sufficient time resolution for tasks involving movement (e.g., walking; Cappellini et al., 2006; Artoni et al., 2013).

Within the MoBI framework a series of recent works have shown that electrocortical dynamics, particularly in the sensorimotor cortex, exhibits intra-stride patterns of activation and deactivation (Gramann et al., 2011; Gwin et al., 2011; Chéron et al., 2012). However, verifying the existence of a true brain-to-muscle link definitely requires the definition of new methodological approaches based on e.g., brain-muscle connectivity measures during this task and in particular the combined use of both EEG and EMG (Petersen et al., 2012; Artoni et al., 2017). Including EMG analysis in the MoBI framework is particularly important to sort meaningful brain activity from EEG artifacts (e.g., cable movements, electrode/gel coupling, nonstationary line noise, movement artifacts) coupled to the gait phases and overlapping in time and frequency with the EEG (Castermans et al., 2014).

Synchronization of different data streams (e.g., EMG, EEG, motion capture camera, eye tracking devices, force platforms, insoles, body sensor networks (Chen et al., 2011; Martelli et al., 2014 etc.), all with different sampling rates (ranging from 50 Hz—simple camera, 2000 Hz—EEG or 40 kHz—Audio), constitutes one of the greatest challenges that restrict the access of the EEG-EMG MoBI framework, especially in clinical settings. In fact, the time constraints imposed by brain imaging—e.g., early somatosensory evoked potentials can appear as early as 20 ms after a tactile stimulus (Genna et al., 2017)—warrant special care in setting up recordings as synchronization delays or synchronization jitter constitute a serious threat to the extraction of meaningful results from MoBI paradigms. Time shifts between single devices, starting from 10 ms, may alter causal links in EEG—EMG connectivity estimates (Kline et al., 2016) and may lead to misinterpretation of the obtained results. In fact Grosse et al. (2002) show that cortical activity precedes EMG by a delay appropriate for conduction in the fast conduction pyramidal pathway (around 15 ms).

Here, after a brief review of strategies for EEG and EMG synchronization, we describe and test a worst-case-scenario fallback solution, that can be used to quantify the jitter, detect possible non linearities, study the effect of hardware filters, and estimate a safe length of recording sessions to achieve MoBI-grade synchronization. We also show why synchronization cannot rely just on the equipment sampling rate advertised by manufacturers and on simple alignment to the start of the recording.

## Materials and Methods

### Overview of EEG and EMG Synchronization Strategies

**Figure 1:**
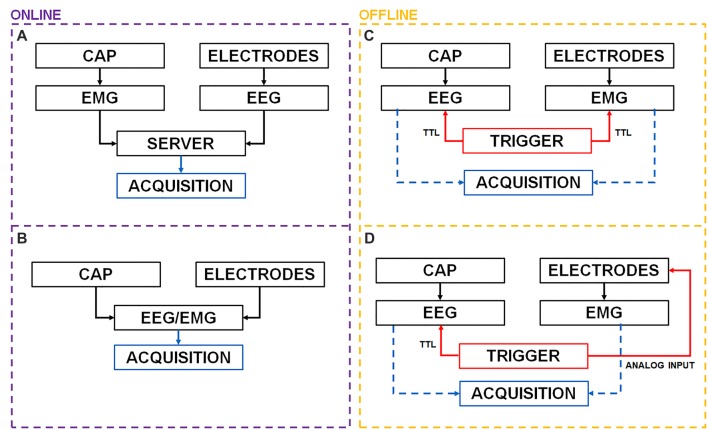
Possible offline and online electroencephalogram (EEG) and electromyogram (EMG) synchronization architectures. **(A)** EEG and EMG data are pushed sample by sample or chunk by chunk to a server that timestamps and merges multiple streams. The server may then forward the data over a lab network or store it for offline use. **(B)** EEG electrodes are detached from their holder and used to record EMG data. Samples are automatically synchronized at collection time. **(C)** If both EEG and EMG amplifiers provide a Transistor-Transistor-Logic (TTL) port simultaneous digital pulses may be delivered to perform offline synchronization. **(D)** In case a TTL port is not available analog pulses may be directly delivered to an EMG electrode before and after the recording session.

Both EMG and EEG devices comprise a set of electrodes (wired or wireless) fixed to a subject via a cap (EEG) or electrode holders (surface EMG), an AD amplifier that delivers sampled, highpass filtered (or DC-filtered) signals to a device (e.g., server, PC) that collects and stores data on durable supports (hard drive or SD card). Figure 1 shows several possible synchronization strategies, in particular panel A shows the current state of the art solution to handle real time EEG and EMG synchronization.

The EEG and EMG amplifiers collect the data from electrodes. Cap (EEG electrodes) and EMG electrodes may be connected to their acquisition devices either via cable or wirelessly. Data are then made available as time series streams over a network and pushed sample by sample or chunk by chunk to a server that handles real-time acquisition and synchronization. Each data sample is associated with a timestamp by the recording device and different streams can then be synchronized by the server and resampled to the desired frequency either offline or online. Lab Streaming Layer (LSL; Kothe, 2014) is an open source data acquisition project that relies on clock offset measurements to handle event information and timing as well as synchronization across devices capable of delivering a data stream output (Reis et al., 2014). Data can then be transmitted in pseudo real-time (i.e., with delay depending on network latency) to other devices e.g., over a network. This approach requires reliable and timely transmission of data packets over the network. On some devices (pressure foot insoles or HD-cameras), battery and high bandwidth requirements may not allow reliable, consistent and timely transmission of data packets over the network. Data can be also recorded locally (e.g., over a SD-card) when network stability cannot be guaranteed or when there is a risk of falling out of communication range (especially with short range communication such as Bluetooth) and data packets may be lost. Unfortunately, LSL requires access to Application Programming Interfaces (APIs), availability of a continuous data stream or requires supported hardware. APIs are however seldom available in clinical settings, e.g., due to device certification requirements. Another solution is that of using, if available with the EEG equipment, extra bipolar channels for EMG measurement (Figure 1B).

A fallback solution (Figure 1C) is to use a common input (spikes) simultaneously sent to all devices e.g., through a Transistor-Transistor-Logic (TTL) port. Before starting the experiment session an external trigger may deliver a digital input (i.e., analog square wave pulse compliant with TTL specifics for voltage: [0–0.5 V] for LOW and [2.7–5 V] for HIGH) to multiple devices.

Repeated synchronization pulses should be delivered to all devices throughout the whole experiment to avoid desynchronization and time drifts, however in some situations this may not be possible: for instance, the EMG receiver (with wireless muscle sensors e.g., Noraxon Telemyo DTS) may be fixed in place, while the EEG amplifier (e.g., ANT Neuro eego sports) is carried by the subject during a MoBI session. In this situation, attaching a cable to both can be unpractical. A compromise would be that of delivering pulses both before and after the recording session and use them to align multiple time series. Again, some clinical hardware devices (e.g., BTS Free EMG 300, BTS Free EMG 1000) do not provide a TTL synchronization port or TTL pulses might be delayed by the equipment (e.g., with Noraxon Desktop DTS the advertised delay between EMG measurement and delivery is 72 ms) making this type of synchronization less effective.

Here we describe and test the worst-case scenario fallback solution shown in Figure 1D, that can be used when the aforementioned strategies (Figures 1A–C) are unavailable (e.g., when state of the art research-purpose hardware equipment or APIs are not available). This synchronization strategy, called “PRE—POST recording” alignment, is based on the delivery of series of pulses both before and after the recording, sent directly to the EEG TTL port (always available) and to one EMG electrode. Data can then be interpolated and aligned offline to achieve MoBI-grade synchronization.

### Summary

To test the effectiveness of a synchronization strategy based on the delivery of pulses both before and after a recording session we delivered a train of spikes to the EEG amplifier and EMG electrodes every 2 s over a 10-min time period. We selected a variable number of spikes (from 1 to 10) both at the beginning and end of the timeseries and linearly resampled the data so as to align them. We then compared the misalignment of the “middle” spikes over the whole recording to test for jitter and synchronization drifts, highlighting possible nonlinearities (spikes delivered directly on the EMG electrode are filtered by the EMG hardware) and estimated the maximum length of the recording to achieve a safe (for refined MoBI analyses) [−5 to 5] ms misalignment range.

### Experiment SetUp

**Figure 2:**
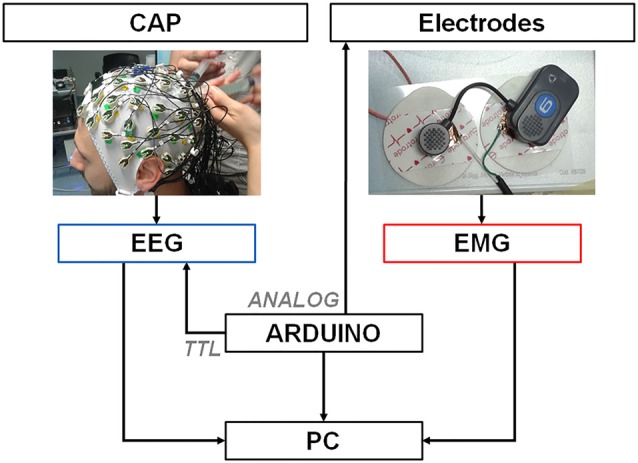
Recording and synchronization platform. A PC is connected through a serial port to an Arduino Zero platform that simultaneously delivers digital TTL pulses (spikes) to the EEG amplifier and analog 3 mV – 4 ms pulses to one EMG electrode by means of a custom-designed cable. EEG and EMG signals collected by the electrodes are transmitted to the respective amplifiers that deliver the data to a PC for later offline synchronization.

The experiment was carried out at Villa Beretta—Ospedale Valduce, LC, Italy with a EEG Neuroscan SynAmps2 and the BTS Free EMG 1000. Neither APIs nor TTL input synchronization ports were available for the EMG. Figure 2 shows the experiment setup. The subject gave his written informed consent to the experiment and the protocol was approved by the Comitato Etico Interaziendale delle Province di Lecco, Como e Sondrio. The recordings were carried out in agreement with the Declaration of Helsinki.

We built the synchronization platform by means of an Arduino board, which provided one 10 bit Digital to Analog Converter (DAC) output pin and a 48 MHz clock speed (Banzi and Shiloh, 2014). The Arduino board was connected through a USB cable to a PC via the “programming” port. Commands were delivered to Arduino via the serial port of the computer through a Python interface. Once the command was received, two synchronized signals were generated by Arduino. A TTL trigger pulse was delivered to the Neuroscan EEG amplifier and annotated as “event” by the proprietary recording software. At the same time a 4 ms square wave pulse (spike) with 3.2 mV amplitude was delivered directly to a EMG electrode through a custom cable connected to the DAC output and the ground pins. We tested for sub-ms synchronization of both Arduino outputs by means of a DAQ card (Labjack T7Pro) with a 2000 Hz sampling rate. EMG disposable surface electrodes were modified to allow for a portion of conductive material connected to the trigger cable to latch to the wireless EMG lead, as shown in Figure 2. Noise was reduced by pressing electrode and lead together using a constant weight during pulses delivery.

### Spike Delivery

Spikes were delivered both to the EEG and EMG devices every 2 s to form a spike train. EEG and EMG data were recorded at nominal sampling rate (1000 Hz). The recording lasted 10 min. The onsets of TTL pulses were automatically interpreted as events by the EEG software and annotated to the exported data with a precise timing with respect to the beginning of the recording. EMG spikes timing was determined offline using an amplitude threshold. The threshold was selected as the minimum value that allowed to recognize spikes and guarantee fair robustness to background noise, i.e., the 99th percentile of the amplitude distribution of the spike-free portions of data. The aim was to characterize spike misalignment throughout the recording with different strategies used in practice, namely: (i) by synchronizing the first and last (up to 10) spikes within the recording; and (ii) using only the first (up to 10) spikes to align the data, i.e., relying on the nominal sampling frequency declared by manufacturers. We also tried different thresholds for spike detection.

### Alignment with First and Last Spikes (“PRE—POST Recording” Alignment)

**Figure 3:**
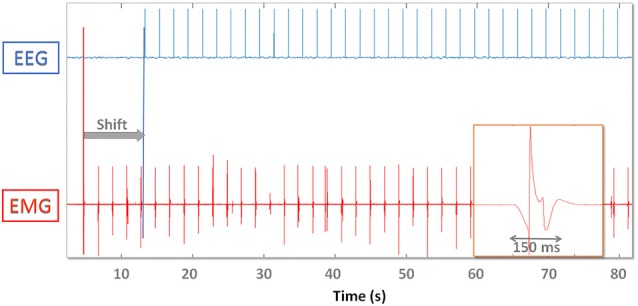
Digital EEG (top) and analog EMG (bottom) synchronization spikes. EMG data are shifted (gray arrow “Shift”) and time warped so that the first and last spike coincide. On the lower right one analog EMG spike is magnified and demonstrates the effect of the hardware analog filter. The EMG spike amplitude and shape varies throughout the recording.

Figure 3 shows the TTL (EEG) and analog (EMG) spike train delivered to devices and the offline synchronization strategy. Ideally if a spike train is available throughout the whole recording it would be possible to interpolate EMG data and align each spike precisely. Here we used the spikes to determine alignment consistency (i.e., stability to synchronization) throughout the whole recording after matching them respectively at the beginning (“PRE spikes”) and end (“POST spikes”) of the recording. This allows to simulate a setup where no wireless connection or extra electrodes are available for synchronization purposes. Given the analog nature of EMG spikes, robustness of synchronization may be increased by aligning the time series to the median misalignment of the first and last n spikes. We used up to *n* = 10 spikes at the beginning and end of the recording respectively and in particular *n* = 1, *n* = 5, *n* = 10.

Within the “PRE—POST strategy” alignment EMG data were first cropped or zero-padded. If *n* = 1 only the first spike was aligned, if *n* > 1 the EMG data were shifted so that the median misalignment across the first n spikes was null. After first cropping, the EMG data were linearly time-warped (resampled). If *n* = 1 the warping was performed so that the last recorded spike of both recordings coincided, whereas if *n* > 1 it was performed so that median misalignment across the last n spikes was null. We then calculated the misalignment between “internal” spikes and computed its density distribution. We then calculated the mean, standard deviation, trend (drift over time) and range. After evaluating Gaussianity (Shapiro-Wilk test, significance *α* = 0.05), we defined as “Jitter” the standard deviation of such distribution. We compared results for each n from 1 to 10 with a one-way analysis of variance (ANOVA; significance *α* = 0.05). We further tested the robustness of the results by repeating the experiment with a longer recording (20 min) and by computing the jitter with *n* = 10 for every subepoch in the 4–20 min range.

### Effect of Threshold on Synchronization (with “PRE—POST Recording” Alignment)

Due to the analog nature of the EMG spike signal (absence of a TTL trigger port) and the effect of hardware filters, single pulses are spread out over up to 200 ms (Figure 3). The timing of the onset of the waveform depends on the threshold used to recognize the spike as an event, which needs to be as low as possible (to avoid delays) but high enough to be robust to background noise activity that might trigger false events. We therefore determined the influence that threshold selection has on results by testing 5% (A), 10% (B) and 20% (C) thresholds (with respect to maximum peak amplitude).

### Alignment with First Spikes (“PRE Recording” Alignment)

Finally, in order to test the reliability of nominal sample rate advertised on EEG and EMG equipment, we determined synchronization stability using the first spikes only. We cropped the EMG data so that the median misalignment of the first 10 spikes was null and determined whether any trend existed. In particular, we calculated the time it took after the recording started, for cumulative desynchronization to reach 20 ms and 60 ms, respectively.

## Results

**Figure 4:**
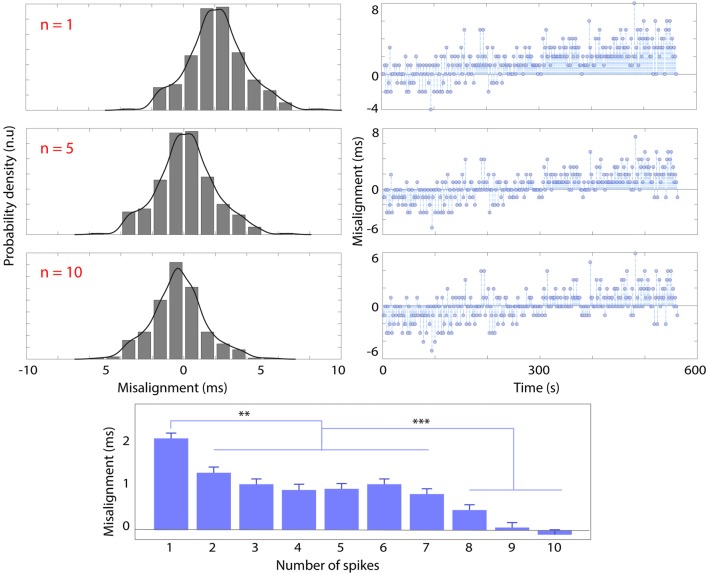
Probability density histogram and kernel-fitted probability density function (left) and trend (right) of spike misalignment throughout the recording (10 min) respectively using *n* = 1 (top row), *n* = 5 (middle row), *n* = 10 spikes before and after the recording session for synchronization. The bottom panel shows the average misalignment (ms) and standard deviation as a function of the number of spikes used for synchronization (n). Significant differences are marked with **p < 0.01 and ***p < 0.001.

Figure 4 shows the overall misalignment distribution (left), with the corresponding trend over time (around 10 min-experiment) using the “PRE—POST recording” synchronization strategy respectively with one (*n* = 1, top row), five (*n* = 5, middle row), 10 (*n* = 10, bottom row) spikes.

The standard deviation of misalignment (i.e., jitter) was not modified by the number of spikes used for PRE—POST alignment (*σ* = 1.7 ms), nor the length of the recording (from 4 min to 20 min). The bottom panel of Figure 4 shows that the average misalignment is inversely correlated with the increase of the number of spikes used for synchronization (*n*). The average misalignment with *n* = 1 is significantly higher (*p* < 0.01) than that obtained with *n* = [2–7], higher still (*p* < 0.001) with respect to *n* = [8–10]. Increasing *n* has the effect of centering the misalignment distribution. The results also show a positive trend of 1.33 * ${\text{10}}^{\text{−5}}\;\frac{\text{ms}}{\text{ms}}$.

**Figure 5:**
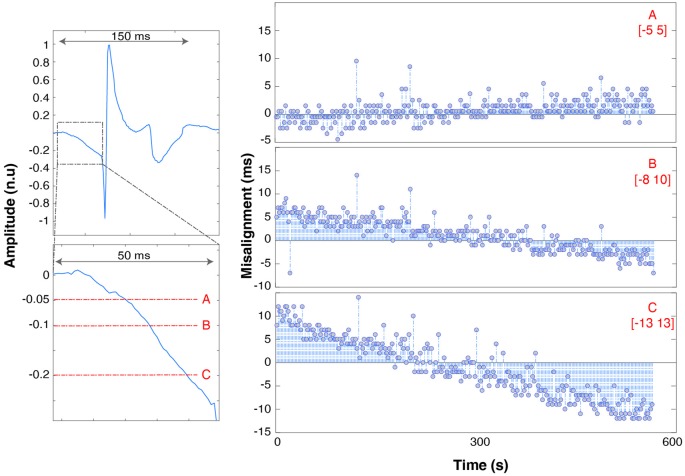
Trend of spike misalignment throughout the recording respectively using threshold “A” (top row, right), “B” (middle row), “C” (bottom row). The range is underlined in red. The left panels show the full spike waveform (top left panel) and a zoom of the spike onset with 5%, 10% and 20% thresholds superimposed in red. The spike amplitude is shown with units normalized to the maximum waveform amplitude.

The effects of EMG hardware filters on spikes delivered are shown in Figure 5 (top left panel).

Spikes are spread over a time span of 150 ms. The onset is marked by a slow descent of the voltage lasting 50 ms before dropping to −1 and bouncing back to +1 (normalized units). It takes 150 ms after spike delivery for the recorded voltage to return to baseline values, which limits maximum spike delivery frequency for synchronization purposes to 5 Hz. Increasing the threshold has the effect of also increasing the jitter and misalignment range: [−5 5] ms for threshold A, [−8 10] ms for B (trend ~3 * ${\text{10}}^{\text{−5}}\;\frac{\text{ms}}{\text{ms}}$ and [−13 13] ms (trend ~4 * ${\text{10}}^{\text{−5}}\;\frac{\text{ms}}{\text{ms}}$) for C.

**Figure 6:**
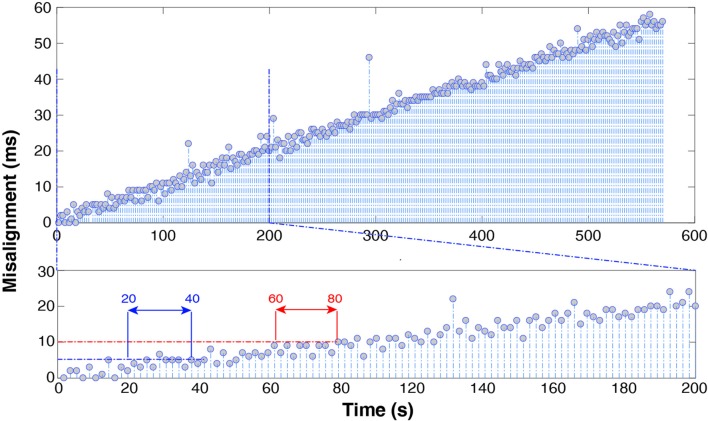
Spike misalignment trend throughout the whole recording (top) and over the first 200 s (bottom) after alignment of EMG relying on manufacturer-declared sampling frequency. It takes only 1 min to accumulate a 10 ms delay.

Figure 6 shows the results using the “PRE—recording” alignment strategy (with *n* = 5). The trend is almost linear and its value is 10 * ${\text{10}}^{\text{−5}}\;\frac{\text{ms}}{\text{ms}}$. EEG-EMG misalignment reaches 5 ms after about 20–40 s into the recording and 10 ms after 60–80 s.

## Discussion

### First-Choice Synchronization Strategies

Offline and real time synchronization (Figure 1A) can be effectively achieved by using LSL (Kothe, 2014). This solution should be considered as the first choice for synchronization due to its ease of use and robustness (plus, the LSL project is open source, thus benefiting of a wide community support). However, this solution cannot be used if the hardware is not accessible (i.e., unavailability of APIs).

If the interest lies only in EMG and EEG, an easier solution than LSL for non-moving subjects, is represented in Panel B, Figure 1. Some EEG devices already account for up to 16 external bipolar EMG channels (e.g., ANT Neuro eego sports), that are already synchronized at hardware level. This solution however may not be practical for MoBI experiments: passive electrode systems are hindered by cable movement artifacts, and cables attached to electrodes (active as well as passive) might get tangled thus impairing a movement (MoBI) task e.g., walking (Reis et al., 2014). In fact, cable-free active-electrode EEG solutions are not yet available: EEG electrodes, even in wireless systems, are designed to be fixed to a cap that collects the cables and holds them in place and are not designed to be detached and fixed e.g., to the legs. Electrode cables are physically connected to a wireless transmitter (see g.Nautilus from g.tec or MOVE by Brain Products for example) that then delivers signals to a receiver. In fact, integrating an amplifier and wireless transmitter within the EEG electrodes themselves would: (i) increase the cost of the EEG device and (ii) increase the weight on the user’s head. While the extra weight is acceptable for EMG electrodes designed to be worn on peripheral muscles, the EEG comes with different specifics. For these reasons current MoBI solutions rely on different amplifiers and systems, each designed and optimized for a specific physiological signal to record e.g., EEG, EMG, foot pressure etc. (Gramann et al., 2014).

### Fallback Strategy

In this article we described a fallback synchronization strategy, “PRE—POST recording alignment” which, although suboptimal, is always applicable and enables the confident use of the MoBI approach in any clinical setting. We showed that it is possible to achieve small-jitter effective EEG-EMG synchronization within the MoBI framework even in worst-case scenarios i.e., when some of the equipment: (i) does not come with enough usable bipolar channels; (ii) does not allow real-time recording; (iii) does not provide a TTL synchronization port; and (iv) does not provide APIs. Unfortunately, these scenarios are actually quite common in clinical environments, often due to restrictions and patient safety regulations (medical grade equipment certifications).

### Duration of Recordings

Results indicate that with 10-min recording sessions, it was possible to achieve a *σ* = 1.7 ms jitter with range [−5 5] ms (Figure 4). With the need to maintain a 10 ms maximum misalignment interval between these two measures, researchers can limit the duration of their recording sessions to this time. Although maintaining a 10-min limit for recording is generally advisable for other reasons (e.g., reducing the maximum amount of data lost in case of hardware/software failure), further tests revealed that shorter or longer recordings, up to 20 min, guaranteed a jitter in the [1.3 2.2] ms range. If longer recordings or different systems are needed, the methods hitherto described can be used to effectively test the characteristics of new systems. The 10 ms safety threshold for maximum misalignment derives by the assumption that EEG and EMG connectivity analysis is to be performed. However, if less-sophisticated analyses are required (e.g., time-frequency transformations based on 0.2–0.5 s time windows), the synchronization demands may be more lax.

### Non-linear Phenomena

By using at least two spikes for “PRE—POST recording” synchronization it is possible to better center the misalignment distribution with respect to using only one spike, 9–10 spikes are optimal. However, it can be seen (Figures 4, 5) that even by linearly aligning the time series with 10 spikes, a significant trend in the misalignment values is still present. This is clear indication of nonlinear effects, which can be probably ascribed to many concurring factors such as: (i) background noise and hardware filters that make spike onset detection for the EMG not instantaneous; (ii) small changes over time of the slope of the spike (due to the hardware electronics); and (iii) numerical approximations that might slightly impair linear resampling algorithm performance. To reduce the trend and standard deviation, the threshold should be as low as possible. A template-based spike detection might also be used, although the non-stationarity over time of the spike waveform would probably prevent precise detection (Kim and McNames, 2007). Else, non-linear time-warping can be performed. These results incidentally highlight the importance of always performing a jitter test like the one presented here, as it enables us to detect possible non-linearities, quantify the robustness of the synchronization and determine the appropriate duration of the experiment, based on the requirements.

### On Manufacturers’ Advertised Sampling Rate

The results show that manufacturers’ advertised sampling rate should not be relied upon when performing EEG-EMG alignment. Figure 6 clearly shows that 1 min into the recording it is already possible to appreciate a 10-ms misalignment. In fact, the actual sampling frequency difference between the two devices, required to maintain alignment up to 1 ms over a 10-min recording needs to satisfy the equation (*f*_1_−*f*_2_)*T* < 10^−3^ with *T* = 600 s, which yields Δ*f* < 1.6 * 10^−6^. This precision in advertised sampling rate (order of magnitude 0.000001 Hz), unless specifically stated by the hardware producer, is in practice never provided, as it exceeds common user’s requirements. The trend shown in Figure 6 is proof of this. In short, alignment and resampling are not sufficient to guarantee successful EEG and EMG synchronization, but it is necessary to align spikes both before and after each recording session.

## Conclusion

Here we discussed several synchronization strategies and tested the effectiveness of a technique for synchronizing EEG and EMG data based on the alignment of spikes delivered to both devices, respectively at the beginning and end of the session. We demonstrated that MoBI-grade synchronization can be achieved for 10-min recordings with a *σ* = 1.7 ms jitter and [−5 5] ms misalignment range, which allows to compute brain-muscle connectivity. PRE—POST recording alignment can be safely used in every clinical environment, effectively making the MoBI framework available to virtually any clinic or research lab.

## Author Contributions

FA and AB developed the experiment setup, supervised the experiment, analyzed the data, discussed the results and wrote the article. FA, AB and EG performed the experiment. FA and AL designed the study. SM, AL and FM co-supervised the experiment. All authors approved the final manuscript.

## Conflict of Interest Statement

The authors declare that the research was conducted in the absence of any commercial or financial relationships that could be construed as a potential conflict of interest.
